# *In‐situ* glial cell‐surface proteomics identities pro‐longevity factors in *Drosophila*

**DOI:** 10.1002/alz70861_109037

**Published:** 2025-12-23

**Authors:** Madeline Paige Marques

**Affiliations:** ^1^ University of Texas Health Science Center at Houston, Houston, TX USA

## Abstract

**Background:**

Recently, much focus has shifted towards understanding how glial dysfunction may be contributing to age‐related neurodegeneration. This is largely due to the many crucial roles glial cells play in maintaining healthy brain function, such as moderating cell‐cell interactions, maintaining homeostasis, immune responses, etc. Cell‐cell interactions, which are mediated by cell‐surface proteins, control nearly all aspects of development and physiology; as such, dysregulation of glial cell‐surface proteins in particular is hypothesized to play an important role in age‐related neurodegeneration. However, it remains technically difficult to profile glial cell‐surface proteins in intact brains, where many cell types intermingle within a very compact region.

**Method:**

Recently, we worked alongside our collaborators to develop a platform to profile spatiotemporally resolved cell‐surface proteomes from intact brains in *Drosophila*. Importantly, this enables us to fully profile cell‐surface proteomes in‐situ and preserve important global dynamics and cell‐cell interactions that would otherwise be lost using more traditional proteomics methods. Applying this platform to aging‐model (i.e., young and old) flies, we investigated how glial cell‐surface proteomes change as during normal brain aging.

**Result:**

We identified several candidate genes associated with neural development and wiring molecules not previously thought to be particularly active in glia or in normal brain aging, including DIP‐β, which was associated with significant increases in lifespan when it was constitutively over‐expressed in glia as adult flies aged.

**Conclusion:**

Our study is the first to apply *in‐situ* cell‐surface proteomics to glial cells in *Drosophila*, and fully profile and characterize the glial cell‐surface proteomes of aging‐model (young and old) flies. Additionally, while DIP‐β’s role in precise neural circuit assembly during development has been studied extensively, ours is the first to identify DIP‐β as a potential regulator of healthy brain function and aging in adult flies.

